# Liver Transplantation in the Presence of Old Portal Vein Thrombosis

**Published:** 2010-02-01

**Authors:** F. Kakaei, S. Nikeghbalian, H. Salahi, A. Bahador, K. Kazemi, M. Dehghani, A. Shamsaeefar, B. Sanei, S. Ghaffaripour, E. Rajaei, S. Gholami, S. A. Malek-Hosseini

**Affiliations:** *Organ Transplant Research Center, Nemazi Hospital, Shiraz University of Medical Science, Shiraz, Iran.*

**Keywords:** Portal vein thrombosis, liver transplantationl, Iran

## Abstract

Background: Portal vein thrombosis (PVT) has been mentioned as a potential obstacle to liver transplantation (LTx).

Objective: To review the impact of PVT on orthotopic liver transplant (OLT) outcome.

Method: Between January 2006 and April 2009, 440 OLT were performed in Shiraz Transplant Unit of whom, 35 (7.9%) cases had old PVT with recanalization. Data were retrospectively collected regarding the demographics, indication for OLT, Child-Turgot-Pugh classification, pre-transplant diagnosis of PVT, perioperative course and managements, relapse of PVT, early post-operative mortality and morbidity. All patients received liver from deceased donors, underwent thrombendvenectomy with end-to-end anastomosis without interposition graft and evaluated daily for 5 days and thereafter, biweekly by duplex sonography during the follow-up period for 2 months. They were treated by therapeutic doses of heparin followed by warfarin to maintain an INR of 2–2.5.

Results: The causes of end-stage liver disease were hepatitis B in 11, cryptogenic cirrhosis in 11, primary sclerosing cholangitis in 5 and other causes in 8 recipients. Extension of thrombosis was through confluence of superior mesenteric and splenic vein in 32 and to superior mesenteric vein in 3 patients. The mean±SD operation time was 7.2±1.5 hrs. The mean±SD transfusion requirement was 5.4±2.8 units of packed cells. The mean±SD duration of hospital stay in these patients was 17.7±10.9 days. Eight patients died; 1 developed early in-hospital PVT, 1 had hepatic vein thrombosis, and 1 died of in-hospital ischemic cerebrovascular accident, despite a full anticoagulant therapy. The mean±SD follow-up period for those 28 patients discharged from hospital was 16.6±7.9 months; none of them developed relapse of PVT. The overall mortality and morbidity was 28% and 32%, respectively. There was no relapse of PVT in the other patients.

Conclusion: The presence of PVT at the time of OLT is not a contraindication for the operation but those with PVT have a more difficult surgery, develop more postoperative complications, and experience a higher in-hospital mortality.

## INTRODUCTION:

Portal vein thrombosis (PVT) is a complication of chronic liver disease, which occurs in approximately 5%–15% of the patients [1]. At the beginning of orthotopic liver transplant (OLT) history, PVT was considered as an absolute contra-indication to this operation [2], and is still treated as a relative contra-indication in some centers [2]. Screening for PVT is routinely performed in all candidates for OLT, mainly by Doppler ultrasonography. Despite exhaustive radiological evaluation before OLT, cases of undiagnosed PVT continue to be encountered during surgery. Different approaches have been proposed to restore portal vein patency at the time of OLT. These included thrombophlebectomy, portal vein resection with or without venous graft interposition, portal revascularization from the superior mesenteric vein using venous graft, or cavoportal hemitransposition [3-6]. In this study, we reported on our experience with OLT in patients with PVT.

## PATIENTS AND METHODS

Between January 2006 and April 2009, 440 OLTs were performed in Shiraz Transplant Unit, Nemazi Hospital, Shiraz, Iran. Thirty-five (7.9%) patients (26 men, 9 women) were found to have PVT at the time of operation. Their mean±SD age was 37.6±12.4 (range: 2–57) years. Data were retrospectively collected regarding the demographics, indication for OLT, Child-Turgot-Pugh classification, pre-transplant diagnosis of PVT, peri-operative course and managements, relapse of PVT, and early post-operative mortality and morbidity. 

Hepatectomy technique in the transplant recipient did not depend on the presence or absence of PVT. W**e **used the piggyback (vena cava-sparing) technique for all recipients. OLT was performed using a whole cadaveric liver graft in 32 patients, splitted extended right lobe in two and splitted left lateral segment in one aged two years. Using the hilar approach, the portal vein was transected from the liver parenchyma once the liver was ready to be removed. Thrombendvenectomy was done under complete visual control by eversion technique previously presented by Dumortier, *et al* [12]. The portal vein was dissected in its entirety to determine the extent of the old and fibrotic thrombus. Maintaining the portal vein open by using two tonsil clamps, the cleavage plane between the thrombus and the intima was found using an endarterectomy Bengolea dissector. Then, the clot was progressively and circumferentially freed by everting the venous wall. This maneuver was extended to the splenic and/or superior mesenteric veins, if necessary. After the clot had been pulled out, portal vacuity was assessed by introduction of the surgeon’s index finger or a Hegar dilator. This technique allowed the entire clot material to be removed. Before completing the anastomosis, the blood flow in the recipient portal vein was verified by removing the clamp. The portal vein was flushed with blood in order to eliminate residual or newly formed clots. Subsequently, the portal flow was restored by end-to-end portal anastomosis.

Follow-up of our patients consisted of Doppler ultrasound daily at first in the five post-operative days followed by two months of biweekly examination. Thereafter, we examined patients when their liver enzymes rose over three times or their clinical condition deteriorated by any means. The patients were treated with therapeutic doses of heparin followed by warfarin to maintain an international normalized ration (INR) of 2–2.5 for at least six months after the operation.

## RESULTS

The incidence of PVT at the time of OLT was 7.6% (35 of 440). Indications for OLT were hepatitis B (n=11), cryptogenic cirrhosis (n=11), primary sclerosing cholangitis (n=5), and other causes (n=8) including autoimmune hepatitis (n=4), Budd-Chiari syndrome (n=2), alcoholic cirrhosis (n=1), and biliary atresia (n=1). The mean±SD Model for end-stage liver disease (MELD) score was 22.1±4.1; the Child classification was B in 11 and C in 24 patients. The mean±SD cold ischemic time was 7.8±3.3 hrs. The mean±SD donor age was 31.4±12.6 years. The mean±SD operation time was 7.2±1.5 hrs. The mean±SD transfusion requirement was 5.4±2.8 units of packed cells. Ascites was over 3000 mL in 27 patients. The mean±SD duration of hospital stay in these patients was 17.7±10.9 days. PVT was diagnosed pre-operatively in 20 (57%) cases during the last pre-operative imaging evaluations which had been done within three months prior to the operation. PVT was <50% of the lumen in 26 patients and >50% in nine patients. Thrombus extension was through confluence of superior mesenteric vein and splenic vein in 32 and to the superior mesenteric vein in three patients. Portal thrombendvenectomy was successful in all of them in spite of thrombus extension with no need for extension venous graft for end-to-end anastomosis. 

There was one patient with post-operative re-thrombosis (4%); she was a two-year-old female recipient with biliary atresia for which she underwent OLT. She received *in situ* splitted left lateral segment from a 37-year-old deceased man with a total cold ischemic time of two hours. The patient underwent re-operation within the first 24 hrs after the operation. Intraoperative exploration showed complete PVT. Re-thrombectomy was not successful and she died three days later. Four other patients died within the first months: three because of primary non-function, one for hepatic vein thrombosis and another, a 53-year-old woman, died of severe brain stem ischemic cerebrovascular accident (CVA) seven days after the operation with a completely functioning liver graft.

The mean±SD follow-up period for those 28 patients who were discharged from hospital was 16.6±7.9 months; none having relapse of PVT. Another patient died of resistant chronic rejection after 43 months. The overall mortality rate was 22.8% out of which 8.6% was due to thrombotic complications. The overall morbidity was 28.5% including three patients with transient renal failure (urine output <0.5 mL/kg/hr) who required continuous renal replacement therapy (CRRT), two who needed re-operation due to bleeding from suprahepatic vein anastomosis, one who underwent reoperation for bleeding from Roux-en-Y distal anastomosis and two patients who developed wound infection.

## DISCUSSION

PVT is a complication or a cause of chronic liver disease. In early series, the reported incidence of PVT in cirrhotic patients was reported as 0.6%–64.1%, and highly dependent on the diagnostic methods used [7-9]. The screening technique most often used for imaging the portal vein is now Doppler ultrasonography [10, 11]. In selected cases, referred for OLT, this incidence is lower ranging from 2.1%–26% [4, 5, 7, 12-18]. PVT was initially considered as a contraindication for OLT [4], and operative mortalities directly attributable to PVT were reported in the first case series in early 1980s [2]. Despite surgical progress in the 1980s, the perioperative mortality rates remained high in the presence of PVT, ranging from 9.1%–42% [4, 5, 13-15] which occurs in approximately 5%–15% of these patients [1].

Factors found to be significantly associated with PVT occurrence include male sex, spontaneous porto-systemic shunts, previous treatment for portal hypertension including endoscopic therapy, transjugular intrahepatic porto-systemic shunt (TIPS), shunt surgery, splenectomy, Child-Pugh class C, alcoholic liver disease, repeated episodes of encephalopathy, severe ascites, Budd-Chiari syndrome, hypercoagulable states, and cancer [3, 13, 17, 19-21]. In our series, 16 (64%) patients had Child-Pugh class C, one (4%) had Budd-Chiari syndrome, and 17 (68%) had severe ascites.

Recanalization of the portal vein was often presented with a thickened and fibrotic vein wall. Portal hypertension was often severe in patients with considerable compromise of portal lumen, which was presented with very huge collateral veins in abdominal wall, retroperitoneum and porta hepatis. 

Different surgical procedures have been proposed during the last 15 years for the surgical approach to treating PVT during OLT. Resection of the portal vein led to porto-portal anastomosis with or without an iliac venous graft obtained from the donor [4]. If the thrombus extends to the splenomesenteric confluence, restoration of portal perfusion may be obtained by anastomosing the donor portal vein to the superior mesenteric vein [3, 4], to the splenic vein [24], to the left gastric vein [3, 25], to choledochal [13, 26, 27], or to mesenteric varices [28], with or without an iliac venous graft. However, the results of these complex surgical procedures are poor [14, 16, 29]. In some exceptional cases, anastomosis between the donor portal vein and the recipient splanchnic system is not possible. This is the place for cavoportal hemitransposition [30]. An alternative procedure is to use a spontaneous splenorenal shunt to obtain a portal flow to the liver graft. Obstruction of the portal tract must probably be relieved, preferably by eversion thrombendvenectomy [12], which represents the simplest way to restore portal flow. This might be difficult in the case of old, extensive or complete PVT. In our experience, thrombectomy with good portal flow restoration was possible and successful in all patients with subsequent end-to-end anastomosis with no need for a venous graft. We believe that eversion thrombectomy is the method of choice to obtain an optimal portal flow, and other procedures must be limited to the failure of simple thrombectomy and usual end-to-end portal anastomosis. 

It has been reported that OLT in patients with PVT is associated with a higher rate of complications, such as hepatic artery thrombosis, re-laparotomy, pancreatitis, sepsis, and renal failure [17]. Moreover, the operation time could be longer and transfusion requirements could be higher [3, 5, 16, 17]. A higher incidence of primary nonfunction (PNF) or dysfunction could be associated with a more complex surgical procedure in patients with severe portal hypertension [17]. We had three (8.5%) patients with PNF in our series, as compared to an overall PNF rate of 9.6% during the same period. Re-thrombosis of the portal vein has been reported in up to 28.5% of patients [13, 14, 16, 29]. In our series, we had only a young patient with portal vein re-thrombosis and two who developed thrombus-formation related complications (one with hepatic vein thrombosis and another with ischemic CVA). Even though clinically evident late re-thrombosis was not documented in our series, some patients could still have developed re-thrombosis that the follow-up studies failed to detect.

According to recent reports [17, 31], mortality rate in the case of re-thrombosis is 100%, similar to our series. Although some authors recommend no systematic post-operative anticoagulation therapy, aspirin, low molecular weight heparin, dextran, and warfarin and its derivatives have been used [14, 16]. Although the role of these prophylactic measures remains unclear, we used a combination of early heparin followed by late warfarin therapy. We encountered five bleeding episodes presented as intra-abdominal hemorrhage in four patients and gross hematuria in one due to warfarin overdose during the follow-up with no need for re-operation. The other three patients were all due to surgical causes. 

## CONCLUSION

The presence of PVT at the time of OLT is not a contraindication for OLT. However, patients with PVT need a more extensive surgery, have more post-operative complications, and higher in-hospital mortality rate.
